# “Money is everything. If you don’t have cash, no one will come to you:” investigating the relationship between socioeconomic status and HIV risk among adolescents and young adults in Kisumu, Kenya

**DOI:** 10.1186/s12889-026-26975-4

**Published:** 2026-03-18

**Authors:** Juliet Fang, Kawango Agot, Nyawira Gitahi, Jane Moraa Arasa, Margaret W. Gichane

**Affiliations:** 1 Brown University, 69 Brown Street Box 2979, Providence, Rhode Island 02912 USA; 2 Impact Research and Development Organization, Kisumu, Kenya; 3 Kenya Medical Research Institute, Nairobi, Kenya; 4 Department of Obstetrics, Gynecology, and Reproductive Sciences, University of California San Francisco, San Francisco, USA

**Keywords:** HIV, Kenya, Adolescents, Young adults, Wealth, Risk

## Abstract

**Background:**

Previous studies have reported a positive association between socioeconomic status (SES) and HIV infection in sub-Saharan Africa (SSA). This contradicts widespread understandings of how health disparities are created and sustained through socioeconomic disparity. The nature of high HIV prevalence in SSA among wealthier individuals, especially those aged 15–24 who experience wealth and HIV risk differently than other age groups, is less understood. Qualitative investigation can provide contextual “how” and “why” factors into the relationship between wealth and HIV in this population, aiding intervention and policy efforts.

**Methods:**

We used data from the Adolescent Sexual Health and Economic Study, which comprised of in-depth interviews with 25 adolescents and young adults (AYA) aged 15–24 years residing in Kisumu County, Kenya. AYA were asked to explain their understanding of high and low social standing and describe the social standing of their peers, a measure encompassing socioeconomic status, social image, access to resources, and material possessions. AYA then described their peers’ sexual relationships and what they believed to be the relationship between social standing and sexual behaviors. Data was analyzed using thematic analysis.

**Results:**

Transactional sexual relationships were a common experience for all AYA in the study. Notably, wealthy young men and women described engaging in transactional relationships more frequently than their less wealthy counterparts. While wealthy young men engaged in transactional sex to boost or maintain social status, wealthy young women sought money out of their transactional relationships to buy luxury items they could not afford with their current supply of money and lack of financial independence. Although less wealthy women also experienced transactional sex, they often did so to obtain money for food and shelter. Participants reported a high awareness of HIV in the community but few of their peers utilized HIV-preventative measures beyond condoms.

**Conclusion:**

Research efforts and interventions cannot overlook groups who traditionally have not been the focus of HIV prevention and management, such as wealthy young men and women. The HIV epidemic in SSA is complex among AYA and cuts across gender and socioeconomic classes.

## Background

Poverty is globally understood to be one of the major factors which contribute to inequities in human immunodeficiency virus (HIV) infection, with wealth being a protective measure against infectious disease [1]. However, in sub-Saharan Africa (SSA), there exists a positive association between socioeconomic status (SES) and HIV infection, contradicting key understandings of how health disparities are created and maintained. Many studies have found that wealthier individuals and wealthier countries in SSA experience a higher HIV prevalence than their poorer counterparts [2].

Specifically in Kenya, a SSA country with one of the highest HIV prevalence rates (5.1%), the highest HIV prevalence rate occurred among the top wealth quintile (7.2%), while the second highest HIV prevalence rate occurred among the second lowest wealth quintile (6.8%) [3]. Those in the lowest wealth quintile were the least likely income group to be living with HIV (4.6%) [3]. One possible explanation for this “paradox” is the informal exchange of money and gifts for sex between wealthy individuals, larger numbers of nonmarital sexual partners compared to poorer counterparts, and lack of condom use during these transactions. These transactional sexual relationships have been observed in SSA for both poor men and women. For women, sex work and transactional sex has been consistently found to be associated with HIV acquisition [4]. Similarly, young migrant men may put their health at risk by getting into sexual relationships out of economic desperation. In Southern Uganda, dating older, wealthier women allowed several young migrant to establish themselves financially in a country where it may otherwise be difficult to gain economic footing [5]. It has also been suggested that wealthier individuals travel more frequently and have increased access to resources for treatment and management of HIV, but the positive relationship between SES and HIV infection is still poorly understood [6].

It has additionally been observed that individuals aged 15–24 in SSA experience a much greater risk of HIV infection compared to other age groups [7]. Of the approximate 6000 new HIV infections that occur worldwide daily, two out of three occur in SSA, with young women bearing a disproportionate burden of these infections. This disparity may be due to earlier sexual debut, age disparate sex, transactional sex, inability to negotiate condom use, and multiple concurrent partners among young women and girls [7]. In 2016, an estimated 133,455 adolescents were living with HIV in Kenya, with 18,004 new infections and 2,797 deaths among adolescents 10–19 years old. Additionally, access to HIV testing, counselling, and antiretroviral therapies by adolescents was significantly lower than for any other age group of persons living with HIV [8]. Previous studies in Kenya have also found that although approximately half of adolescents are aware of HIV prevention methods such as PrEP and were willing to use them, inadequate access to care and low HIV prevention education resulted in underutilization, especially in resource-limited areas [9]. Thus, adolescents and young adults (AYA) are a priority population for HIV intervention.

This study aims to advance the current literature on the relationship between SES and HIV infection in SSA. Current studies are epidemiological in design, missing potential contextual “how” and “why” explanations for the positive association between wealth and HIV infection that can be achieved through qualitative investigation [10]. Further, previous studies have primarily used more objective measures of socioeconomic status, such as income or education. However, subjective measures such as social standing have been found to be more holistic, accurate indicators of health outcomes [11]. Thoroughly investigating this relationship holds great importance for researching and implementing targeted intervention efforts in Kenya, which have historically focused on lower income groups and may be missing opportunities to prevent HIV infection [12]. The key hypothesis for many economic interventions in Kenya is that improving SES and access to resources, income, and education, will reduce engagement in transactional sex, and HIV infection [13]. However, this assumption may overlook certain behaviors of wealthier SSA individuals and their unique HIV risk environment.

There are limited studies which have qualitatively investigated the relationship between diverse social standings and HIV risk behaviors in Kenya, especially amongst AYA, the most vulnerable age group for HIV in Kenya [14]. Understanding the sexual patterns and economic contexts of this population will help inform future research, targeted intervention, and policy efforts.

## Methods

### Study context

This study’s data comes from the Adolescent Sexual Health and Economic Study (ASHES) study, a qualitative study aiming to understand how AYA and parents and caregivers of AYA in Kisumu, Kenya perceive socioeconomic status (SES) and its relationship to sexual behavior among AYA. Kisumu is in the Western Lake region, where over a third of the residents (35.6%) are living in poverty [15] and a quarter of AYA ages 15–24 years are reported as inactive in the labor force (e.g. not actively seeking work, enrolled in school, engaged in care work, or unable to work due to a disability). Nationally, 84% of the Kenyan population work in the informal sector in which individuals are primarily self-employed and work in areas such as trades, food services, agriculture, and transportation. 19% of women and 21% of men ages 15–49 years have more than a secondary education. In 2018, Kisumu County had the second highest HIV prevalence rate in Kenya (17.4%), with an incidence of 6.9 cases per 1,000 person-years [16].

Lack of awareness of HIV status, gaps in linkage to preventative healthcare, and behavioral factors are potentially key drivers of HIV transmission. The current study focuses on these HIV risk factors for AYA with additional insight from caregivers, as older individuals may have more global perspectives of AYA HIV risk environment and behaviors.

### Participants and procedures

This qualitative study collected data through in-depth, semi-structured interviews with a cohort of 25 participants aged 15–24 years and a cohort of 10 caregivers residing in Kisumu County, Kenya. Caregivers were characterized as participants that identified themselves as the primary caregiver to a child aged 15–24 years old. Some (*n* = 2) caregivers were parents or guardians of the AYA participants.

A theoretical sampling technique was utilized to recruit participants. An initial sample of 6 AYA participants and 2 caregivers distributed by age and gender were recruited, and additional participants were recruited and interviewed based on emergent findings. As the primary purpose of this study was to understand perceptions of SES and sexual risk behavior, our recruitment approach was sensitive to local markers of SES to allow us to recruit a socioeconomically diverse sample. Some of these markers included the neighborhoods potential participants lived in or the type of school AYA attended (e.g. day school vs. boarding). An example of an emergent finding which influenced subsequent recruitment included differences in perceptions of SES based on participant residence (rural vs. urban) which led to recruiting a balance of participants across locations. Participants were recruited through fliers, word of mouth, and dialogue with research staff at locations AYA and caregivers frequent. In a few instances, study participants referred friends who had characteristics that the study was looking for. To get a socioeconomically diverse sample, higher SES participants who were harder to reach were recruited through the social networks of research staff.

### Data collection

In-depth interviews were conducted by four research assistants (Jane Moraa Arasa, Lillian Akoth, Nancy Ounda, and Philip Odote) trained in qualitative research methods and fluent in the study setting’s local languages (Kiswahili and Dholuo) and English. The participants first completed a demographic questionnaire and then were interviewed according to a guide with a pre-determined set of questions. Confidentiality was ensured by conducting interviews in a private, quiet location with minimal distractions or interruptions and assigning participants an anonymous study ID. All interviews were audio-recorded, transcribed verbatim, and translated into English.

The interview guide covered topics including peer and personal social standing, sexual relationships, and HIV and pregnancy prevention. AYA were asked to name peers they perceived as high social standing (HSS) and low social standing (LSS), according to their own interpretations of social standing. AYA were also asked to define themselves as HSS or LSS. Social standing encompassed socioeconomic status, social image, access to resources, and material possessions. AYA then shared about their peers’ sexual behaviors and what they believe to be the relationship between social standing and safe or unsafe sexual behaviors.

Caregivers were asked to discuss characteristics of children aged 15–24 they perceived as HSS and LSS and the sexual behaviors of both groups.

### Data analysis

JMA, LA, NO, and PA also transcribed and coded data using Dedoose software. An initial codebook was developed based on a review of interview transcripts, notes, and the interview guide. New codes were developed and discussed among the research team. All transcripts were double coded by two research staff, refined, and then finalized for analysis.

A thematic analysis was conducted by JF, MG, JMA, KA, and NG on the resulting coded excerpts to investigate patterns between social standing and sexual behavior amongst AYA. Analytical memos and matrices were developed to compare major themes within and across participants. Extracted themes were then discussed and finalized.

### Author(s) position statement(s)

The collaborative, analytical approach of this study was enriched by the lived experience of research team authors and assistants with first-hand experience in the study setting of Kisumu, Kenya. Research team members who gathered data in Kisumu were also engaged in data analysis and confirmed the interpretations of the data. This approach ensured that the translation and interpretation of the transcripts accurately and respectfully captured the participants’ experience.

### Ethical approval

The University of California San Francisco Institutional Review board and the Maseno University Ethics Review Committee all approved this study as minimal risk for all participants, including minors. All study participants provided written informed consent and parental consent and assent for minor participants to participate in this study. The study was conducted in accordance with the guidelines of the Declaration of Helsinki. 

## Results

The median age of AYA participants was 19 years (IQR: 17, 21) (Table 1). Approximately half of participants identified as male. Most of the participants identified themselves to be of low social standing and in a non-marital relationship. About 36% of participants were employed and 48% reported having a caregiver employed in the formal sector. Among caregiver participants, the median age was 40 years (IQR: 39-42) and over half of participants had some secondary education or higher (60%) (Table 2).

**Table 1:** Characteristics of AYA study participants (n=25)

	n (%)
Sex	
Male	12 (48%)
Female	13 (52%)
Age (Median, IQR)	19 (17-21)
Education	
Primary	4 (16%)
Secondary	15 (60%)
College or University	6 (24%)
Relationship status	
Single	8 (32%)
Not married, in relationship	13 (52%)
Unknown	4 (16%)
Employed	
Yes	9 (36%)
No	16 (64%)
Primary caregiver	
Parent	20 (80%)
Aunt/Uncle	2 (8%)
Sibling	1 (4%)
N/A	1 (4%)
None	1 (4%)
Primary caregiver's employment type	
Formal	12 (48%)
Informal/Self-employed	10 (40%)
Unemployed	2 (8%)
N/A	1 (4%)
Location	
Rural	10 (40%)
Peri-urban	5 (20%)
Urban	10 (40%)

**Table 2:** Characteristics of caregiver participants (n=10)

	n (%)
Sex	
Male	5 (50%)
Female	5 (50%)
Age (Median, IQR)	40 (39-42)
Education	
Primary	4 (40%)
Secondary	1 (10%)
College or University	5 (50%)
Relationship status	
Single	2 (20%)
Married	8 (80%)
Employed	
Yes	9 (90%)
No	1 (10%)
Employment type	
Formal	3 (30%)
Informal/Self- employed	6 (60%)
Unemployed	1 (10%)
Location	
Rural	3 (30%)
Peri-urban	2 (20%)
Urban	5 (50%)

Overall, the analysis revealed that transactional sexual relationships were a common occurrence among AYA. Wealthy young men and women were reported to engage in transactional relationships more frequently and had more sexual partners than their less wealthy counterparts. However, wealthier AYA were perceived to be more open about their sexual relationships, perhaps to uphold their reputations. Motivations for engaging in transactional sexual relationships also varied among socioeconomic classes and genders. Importantly, these findings should be interpreted within the context that these reported data are on individual’s perceptions of others in their community. Additionally, the secondhand nature of the interview data may contain limitations in the amount of knowledge peers know about one another.

Below, we delve more deeply into these findings supported by interview transcripts, categorized into several thematic groups. 

### Money as a limiting factor for number of sexual partners for boys and young men

“Money is everything. If you don’t have cash, no one will come to you.” (male AYA, aged 21, HSS)

Our analysis revealed stark differences in sexual relationships across social standings and gender. For male AYA, money was associated with number of sexual partners. Many AYA and caregivers noted that HSS men they knew had “many” or “uncountable” numbers of sexual partners, while LSS male AYA had “one” or “none.” HSS men with multiple sexual partners gave their partners money and gifts and received sex and companionship in return.

Appearance of wealth and social power were described by participants to be attractive qualities for AYA males in Kisumu. These attributes were often reported to be demonstrated through sexual relationships with multiple partners. Several participants discussed HSS men having multiple partners as a typical component of the “rich man” lifestyle. Conversely, men without money did not have the resources to support multiple partners and were more likely to engage in long-term relationships with a single partner of similar lower SES. However, the vast majority of the LSS men in this study were described by AYA and caregivers as having one or zero sexual partners. This concept is illustrated by one participant, who summarizes the relationship between money and number of sexual partners for men:

“Those from a high social standing tend to have more than one partner, so they can provide money and gifts for sex… men who date from a low social standing can only maintain a woman from a low social class they can provide for.” (female AYA, aged 24, LSS)

This quote frames male sexual partnering as a social expression of economic capacity and masculinity. Multiple partnerships appear to function as a visible marker of success, potentially encouraging men of HSS to seek out many partners to solidify their social influence. 

### Transactional relationships across socioeconomic classes for girls and young women

Transactional relationships were reported to be common for LSS women. Several participants described female friends who engage in sex for money, food, and basic supplies for their families:

“If someone lives under low standard, she can yearn to have many partners so that at least she can get material gain. She can buy food and clothes.” (female AYA, aged 19, LSS)

“She has boyfriends who give her support. She would tell me, ‘today I will go and have sex with my boyfriend so that I can get some money to buy soap for my family’.” (female AYA, aged 15, LSS)

In each of the instances above, the individuals engaging in transactional sex are adolescents aged 17-21, indicating the prevalence of transactional relationships amongst very young women and girls. This reflects a global pattern which economic vulnerability, gendered power imbalances, and limited access to educational and employment opportunities shape young women’s entry into sexual relationships. Within this context, transactional sex emerges as a method for meeting basic needs. The concentration of such relationships among adolescents underscores the heightened vulnerability of this age group, for whom age, gender, and economic dependence intersect to limit negotiating power within sexual partnerships.

While this study confirms previous research that transactional relationships are common in SSA among LSS women, a significant finding of this study was that many HSS women were also reported to engage in transactional sex for money. One participant describes this this pattern:

“Interviewer: Do they have partners so that they can get money?

Participant: That is what most girls do

Interviewer: They are rich but they still want more?

Participant: Yes.” (female AYA, aged 23, LSS)

When asked why women of HSS would need to seek more money despite being wealthy, one participant explained that it was often to purchase luxury items:

“Maybe the parent has bought her a dress that didn’t please her. [If] the partners had told her that he was going to give her 500…if she goes to the next one… now she has 1000. Now if all these 10 [partners] give her 500, doesn’t she have a lot of money?” (female AYA, aged 19, HSS)

Additionally, one participant described the need for money to be potentially affect all ages of women.

“If she finds someone who can give money… that is where she will stick. In fact, not just young women, even older women go to young men because he gives money…they just want the money, so it’s not only the girls, even women.” (female caregiver, aged 42, LSS)

The experience of needing to engage in transactional sex across wealth groups and ages for women may indicate a lack of financial and social power for women. Despite being from wealthy backgrounds, HSS women can still experience “relative poverty,” in that wealthy women and girls may not have enough financial power or independence to fully satisfy their needs, and thus feel the need to seek out transactional relationships. For context, in 2017, the World Bank reported that only 37 percent of women in SSA have a bank account compared to 48 percent of men. These data suggest that transactional sex is driven not solely by absolute economic deprivation but by gendered disparities in access to and control over resources, even within higher socioeconomic strata.

AYA and caregivers did not describe transactional relationships in which men were the party to receive money or gifts, possibly indicating that men in Kisumu likely have more options than women to fulfill their financial needs. However, wealthy men were described by AYA to have the most sexual partners overall, demonstrating different driving forces for involvement in transactional relationships across genders. 

### Social influence on perceived self-risk

Participants stated that the behavior and relationship patterns they observed among AYA of LSS and HSS were influenced by their social environment. Societal norms and expectations were described as encouraging individuals to engage in sexual behaviors that they perceived to be risky. One participant described how engaging in a transactional relationship with an older partner was normative:

“And because of society and because of her sister’s influence, she thinks these are the right things that she ought to be doing. If you are not smoking weed, then you are not having fun. If you are not out with boys, then you are boring, you know such a mindset. If you are not having an old man [give] you money, you are not attractive, you know such things, such a mindset.” (female AYA, aged 15, HSS)

Another participant echoed this sentiment acknowledging the role of peers at school:

“Interviewer: Okay what makes her have more than two or more than one [partner]?Participant: I think it’s according to her lifestyle, and the school environment also makes her do such things that she does… I think it’s due to peer pressure from other girls in school.” (female AYA, aged 19, HSS)

The use of sex to conform to conventional norms of masculinity was also common. Male participants felt that HSS men were pressured into having more partners to show off their status. One participant reported competing with friends on how many partners they had:

“I can also tell you that because of peer pressure they compete a lot. I have five girlfriends so when the other guy hears that I have five girlfriends, he will end up having even 10 of them.” (male AYA, aged 17, LSS)

One caregiver described the act of having multiple sexual partners as a male to be a means of gaining social acceptance. Without multiple partners, men may be ostracized:

“If you have multiple partners maybe you are regarded as a bull [champion] or something like that. It’s as if there is something positive that goes with that rather than if you have one…if you have only one [partner] or you don’t have any you may be even told that you don’t belong to this particular group. You are a disgrace to this group.” (male caregiver, 56, HSS)

The above excerpt highlights how social expectations can influence sexual choices, potentially encouraging individuals to engage in unsafe sexual practices, such as having multiple partners. The effect of social pressure to take on more partners may be stronger for males than females. Multiple AYA males reported HSS males who have multiple partners to satisfy a sort of “social image”:

“If you could remember the [television] series ‘SUGA’, I think that is [the lifestyle] he is living. Love, sex, and money, because he has the cash and everything, he influences everyone.” (male AYA, aged 21, HSS)

In this excerpt, the AYA male is discussing a HSS male peer that uses money to satisfy his sexual needs and keep up his image as a wealthy, powerful man with money. 

### Other sexual risk factors

Use of PrEP, PEP, and condoms as preventative measures against HIV did not follow any patterns across genders or social standings. Participants expressed that the vast majority of HSS and LSS individuals they knew used at least one form of protection, with condoms the most common choice.

Individuals with only one or few partners tended to only use condoms. Despite knowledge of HIV among HSS and LSS individuals, use of PrEP was far less frequent, potentially due to lack of access, pill burden, or lack of knowledge about their peers. More AYA described measures to prevent pregnancy rather than HIV transmission.

“There is some hospital that they used to go to. It came to light that people were going to take PrEP. So those ladies used to go pick [up] PrEP, and even of late they still go there so that at least they take PrEP to reduce their chances of getting HIV; because they don’t trust their partners.” (female AYA, aged 19, LSS)

One participant described a LSS female who had “one partner because she [was] scared of HIV and STIs in general” (female, aged 19, LSS), while another participant described a LSS male who abstained from sexual relations because he was “afraid he could be infected with HIV” (male, aged 18, LSS). However, a small number of individuals were described by AYA to use no forms of protection:

“I don’t think she protects herself in any way [because] she always bragged about how there is no need to use a condom, it feels better when you don’t protect, if you use a condom you are a coward, so that type of mentality.” (female AYA, aged 15, HSS)

Despite these exceptions, the majority of AYAs described their peers as highly aware of HIV, although few participants could say their peers used measures to protect themselves against HIV besides condoms, such as PrEP. However, from the caregivers’ perspective, most AYA were described to be primarily concerned about unexpected pregnancy because of sex, not HIV. One caregiver attributed this to the lack of proper knowledge AYA have on the risks of HIV:

“As a parent on the other side we are bad. You see that immediately after a girl begins seeing her menses the mother initiates the daughter into family planning. They only tell them, ‘My daughter, you will get pregnant.’ But they are not telling them about HIV"

“I cannot tell you with certainty that these children have known something like protection. At their age, they may not have information that there is protection. Maybe the percentage using protection is very low compared to the older population.” (male caregiver, 40, LSS)

However, one caregiver discussed how their child, a LSS female, was exposed to sex education at an early age,

“Since [my child] went to school I think sex education is being taught from class four or six and you are told about protection…and I think she should know that if she doesn’t protect herself she is either going to get pregnant or the disease [HIV].” (male caregiver, 40, LSS)

This account highlights the drawbacks of knowledge-based prevention interventions in areas with limited access to HIV prevention measures, such as PrEP. While AYA are aware of the risks of HIV, they may be unable to act upon their knowledge without access to proper resources.

## Discussion

This qualitative study conducted among AYA and caregivers of AYA in Kisumu County, Kenya sought to expand current knowledge on the complex relationship between social standing and HIV risk in SSA. Our study is among the first to qualitatively investigate this relationship among 15 to 24-year-olds in SSA can give us insight into how to prevent infection amongst this young population. This study additionally focuses on how wealthy young men and women, a group often overlooked in HIV research, experience HIV risk.

Analyses of interview transcripts revealed three main findings: First, we found that transactional monetary relationships were very prevalent across genders and socioeconomic classes in the age group 15-24, indicating a need for more targeted research and intervention in this age group. Second, we found that wealthier men and were perceived to have more sexual partners than their less wealthy counterparts, which may lead to risker sex. This finding was consistent with previous studies in SSA that report higher odds of HIV infection among wealthier Africans (10) and a vast body of qualitative literature from this region illustrating how limited finances restrict partnerships for poorer men, inadvertently protecting them from HIV [17]. Third, we found that motivations for transactional relationships differed by social standing and gender. Notably, money and gifts were the primary motivation for women to seek out sexual relationships regardless of their woman’s own wealth, indicating a need for alternate means of financially empowering women to seek money to fulfill their needs. For men, social status or acceptance was often described as a key motivation for maintaining multiple partners.

Our results illustrate the need for combination interventions which more effectively target the gendered and economic drivers of HIV risk among AYA in Kenya. Many structural interventions in SSA are based on the Asset Theory, which examines the role of financial assets on social well-being. The theory posits that the possession of assets increases stability against economic shock, self-efficacy, and social power, meaning individuals in SSA with more assets would be less likely to engage in transactional or condom-less sex for resources [18]. However, despite numerous interventions, such as cash transfer programs in SSA, to decrease transactional sex and HIV transmission, the results of such interventions have been mixed or limited in their benefit [19]. Our findings that wealthier individuals in western Kenya are perceived to have sexual partners than their less wealthy counterparts corroborate this, since wealth was not necessarily associated with HIV protective behaviors in our study population. Specifically for women, the shared experience among those of LSS and HSS to engage in transactional sex in order to fulfill their needs suggests that increasing wealth for women is not enough to prevent HIV infection. To that end, we recommend holistic social protection programs that combine economic empowerment, with incentivization of school attendance and job training among AYA [19]. These interventions should be created alongside community stakeholders to ensure they are contextual appropriate and sustainable. These programs must not overlook female HSS AYA, who were found in our study to engage in transactional sex to fulfill basic needs. Empowering this population requires holistic strategies beyond just increasing wealth, especially for women who are already from wealthy backgrounds. 

There is also a need for interventions to address sexual risk among boys and young men of HSS. HSS males were more likely to have multiple partners and were often providers in transactional sexual relationships. Engagement in these behaviors was viewed to gain social acceptance and fitting into masculine ideals. Gender transformative interventions which to seek change norms about masculinity may help encourage boys and men to adopt safer sexual practices including reducing number of partners and shifting expectations around sex and provision in relationships [20]. 

### Limitations and strengths

Our study’s findings are also limited by the participants’ secondhand knowledge of their peers’ sexual characteristics and behaviors. While utilizing peer knowledge of stigmatized sexual behaviors can yield more truthful results than asking participants about their sexual behavior directly, especially in Kenya where HIV stigma is relatively high, the participants may have also been limited by their knowledge of their peers’ behaviors [21]. Wealthier AYA were also more open about their sexual relationships compared to their less wealthy peers, which may have overshadowed sexual dynamics in LSS individuals. Additionally, men may exaggerate, and females may underreport their number of sexual partners to uphold certain social standards. This was a pattern found in a study in Tanzania, in which single women reported about half as many multiple partnerships as men [22]. These limitations withstanding, using secondhand interview data provides insightful details on perceived community norms and shared social knowledge regarding collectively understood associations between wealth, gender, and sexual risk. Out study adds important context to how wealth influences HIV in AYA, a key population that is difficult to reach for HIV treatment and management.

## Conclusion

The perspectives of the study participants provided meaningful insight into the complex association between social standing and sexual behavior among AYA in Kenya. With our findings, we conclude that increased wealth may increase an individual’s ability to have more sexual partners, increasing HIV risk especially for men. However, lack of wealth, financial independence, and power also influenced women to engage in transactional sex. Considering this, we urge that research and interventions for HIV management in SSA must consider the experience of HIV risk across all socioeconomic and genders. Increasing access to PrEP and implementing social protection programs for AYA that include incentivization of school attendance and job training for all AYA individuals may also be beneficial for HIV prevention in the long run. We recommend that researchers develop and implement HIV interventions for AYA in Kenya with their input, collaboration, and continual feedback over time. Importantly, research efforts and interventions cannot overlook groups who traditionally have not been the focus of HIV prevention and management in SSA, such as wealthy young men and women.

## Data Availability

The participants of this study did not give written consent for their data to be shared publicly. Due to the sensitive nature of the interviews and the risk of re-identification, raw transcripts cannot be made publicly available. Anonymized data may be available upon reasonable request to the corresponding author, subject to IRB approval.

## Abbreviations

HIV: Human Immunodeficiency Virus
SSA: Sub-Saharan Africa
SES: Socioeconomic
AYA: Adolescents and Young Adults
HSS: High Social Standing
LSS: Low Social Standing
